# Removal of a Tracheal Mesh Stent: How I Do It

**DOI:** 10.7759/cureus.32140

**Published:** 2022-12-02

**Authors:** Massimiliano Manera, Vincenzo Patruno, Paolo Vailati, Giuseppe Morana, Ernesto Crisafulli, Giulia Sartori, Nadia Castaldo, Alberto Fantin

**Affiliations:** 1 Department of Medicine, Respiratory Medicine Unit, University of Verona and Azienda Ospedaliera Universitaria Integrata of Verona, Verona, ITA; 2 Department of Pulmonology, S. Maria della Misericordia University Hospital, Udine, ITA; 3 Department of Pulmonology, University Hospital of Udine (ASUFC), Udine, ITA

**Keywords:** bronchoscopy, tracheal stenosis, trachea, stent, rigid bronchoscopy

## Abstract

We illustrate how to remove a stent from the tracheal lumen 12 years after its deployment. Maintaining the stent in situ for a long time degrades the stent materials, making it fragile and very difficult to manipulate. A rigid bronchoscopy approach was chosen for the treatment of this case. We describe the preparation of the intervention and its execution step by step.

## Introduction

Central airway stenosis can occur after the placement of a tracheostomy. The best therapeutic approaches for this entity are still debated and include both surgical and endoscopic treatment, with the eventual deployment of a tracheal stent [1-3]. The adverse events related to tracheal stents include displacement, stent fracture, dysphagia, granulomatous tissue formation and respiratory infections [4]. These complications can lead to potentially fatal consequences.

## Case presentation

**Figure 1:**
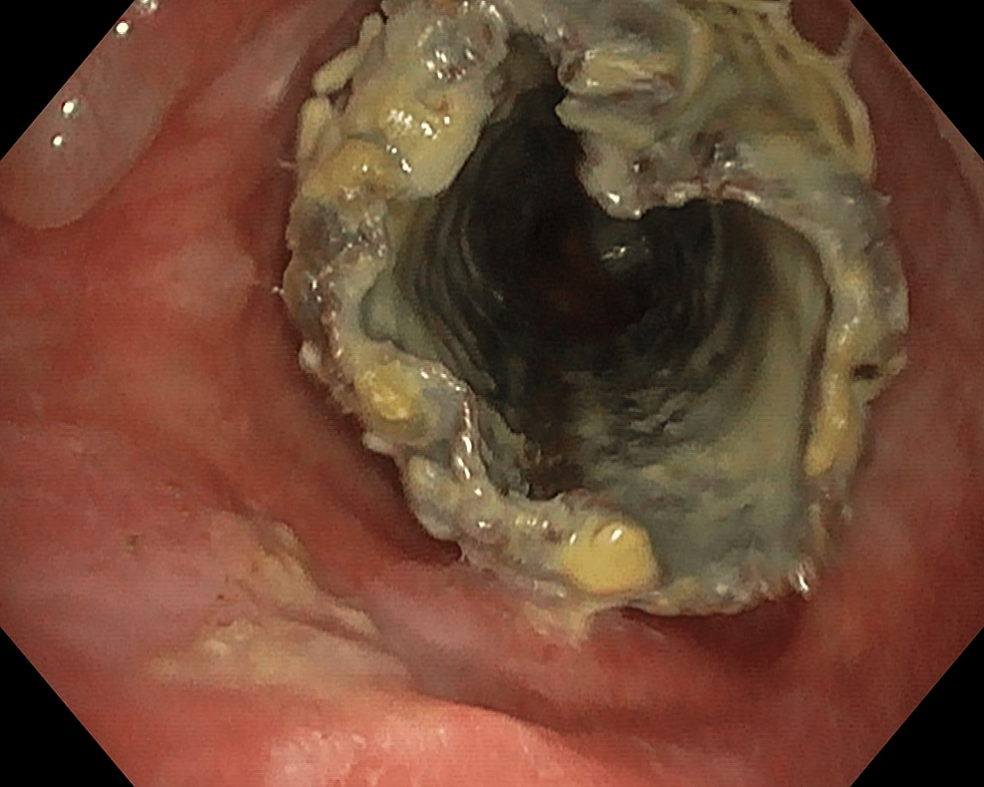
Endotracheal view of the stent, which appears lined internally and externally by greenish secretions.

**Figure 2:**
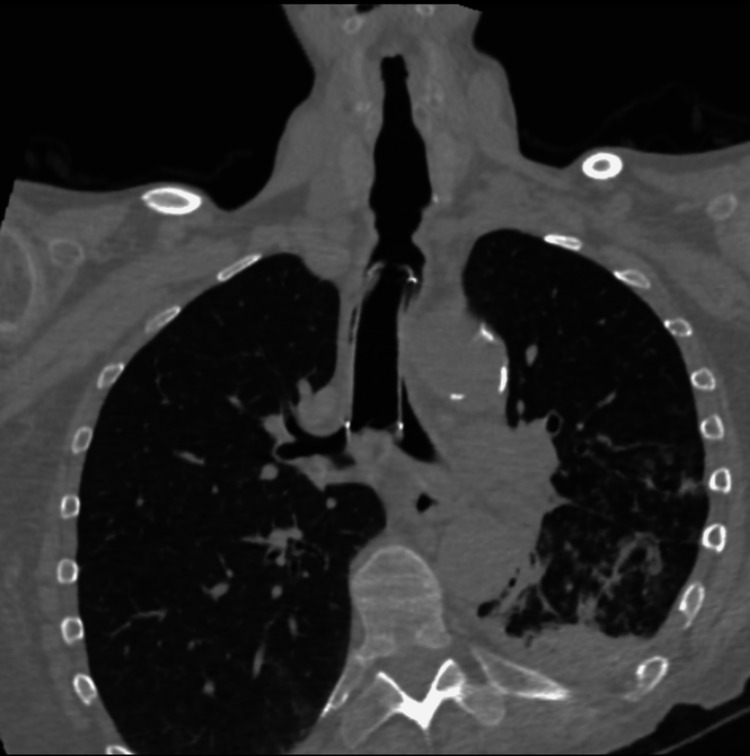
Coronal CT view of the anatomical relationships of the stent.

We present a case of a 46-year-old female patient, who had a clinical history of arterial hypertension and intracerebral hemorrhage, which led to intensive care assistance and the necessity to undergo a tracheostomy for prolonged invasive ventilation. Regarding the tracheostomy tube, she was adequately followed up. Twelve years before her last hospitalization, she was admitted for increased inspiratory effort and wheezing. Due to the high suspicion of a central airway stenosis, an emergency bronchoscopy was performed, revealing a web-like complex circumferential lesion, associated with granulomatous tissue, 2 millimeters below the distal tip of the tracheostomy tube (fourth tracheal ring). The procedure then continued by removing the tracheostomy tube, performing intubation with a rigid bronchoscope, ablating the granulomatous tissue, and, lastly, positioning a tracheal self-expanding stent between the first and seventh tracheal rings: polyester mesh stent, with silicon coating (Polyflex™, Boston Scientific, Marlborough, MA, USA; size: length 60 mm, diameter 18 mm). The patient had a rapid clinical improvement and was discharged two days after the procedure. Five months later, the patient was admitted again for a scheduled endoscopic evaluation, but she categorically refused to provide informed consent to the procedure. The patient was then lost to follow-up, despite the outpatient planning recommended at discharge. She subsequently underwent several outpatient visits to other healthcare institutions for years due to reported halitosis, worsening dysphagia, and recurrent pulmonary infections. She was finally evaluated again at our outpatient clinic due to the development of a new respiratory infection and was admitted. She underwent flexible bronchoscopy (Figure 1), during which a distal migration of the stent placed 12 years earlier was found, being the proximal tract localized at the level of the 6th tracheal ring and the distal tract resting on the carina. The internal lumen of the stent was coated with whitish, dense secretions. The ostia of the main bronchi were partially obstructed by granulomatous lesions originating from the decubitus of the stent on the carina, leaving patency of the lumen equal to 70% on the right and 50% on the left. Abundant greenish secretions were observed. Given the endoscopic evaluation and the history of recurrent infections, removal of the stent was scheduled. Prior to the procedure, the patient underwent a computed tomography (CT) of the chest (Figure 2).

The measurements of the airway lumen on the axial, coronal and sagittal axes were carefully evaluated, in order to predict which instrumentation to use and the possible size of a stent to be deployed in place of the old one. The relationships of the old stent with the tracheal and bronchial wall were inspected. Tracheal and bronchial fistulas with neighboring organs were actively searched for without finding any direct or indirect suspicious signs, and, last but not least, the relationships between the work area and the large mediastinal vascular structures were thoroughly studied in order to know the location of critical areas which needed particular attention during the procedure, as for the grade of pressure applied by the rigid bronchoscope and the energy delivered by the laser beam during ablation.

**Figure 3:**
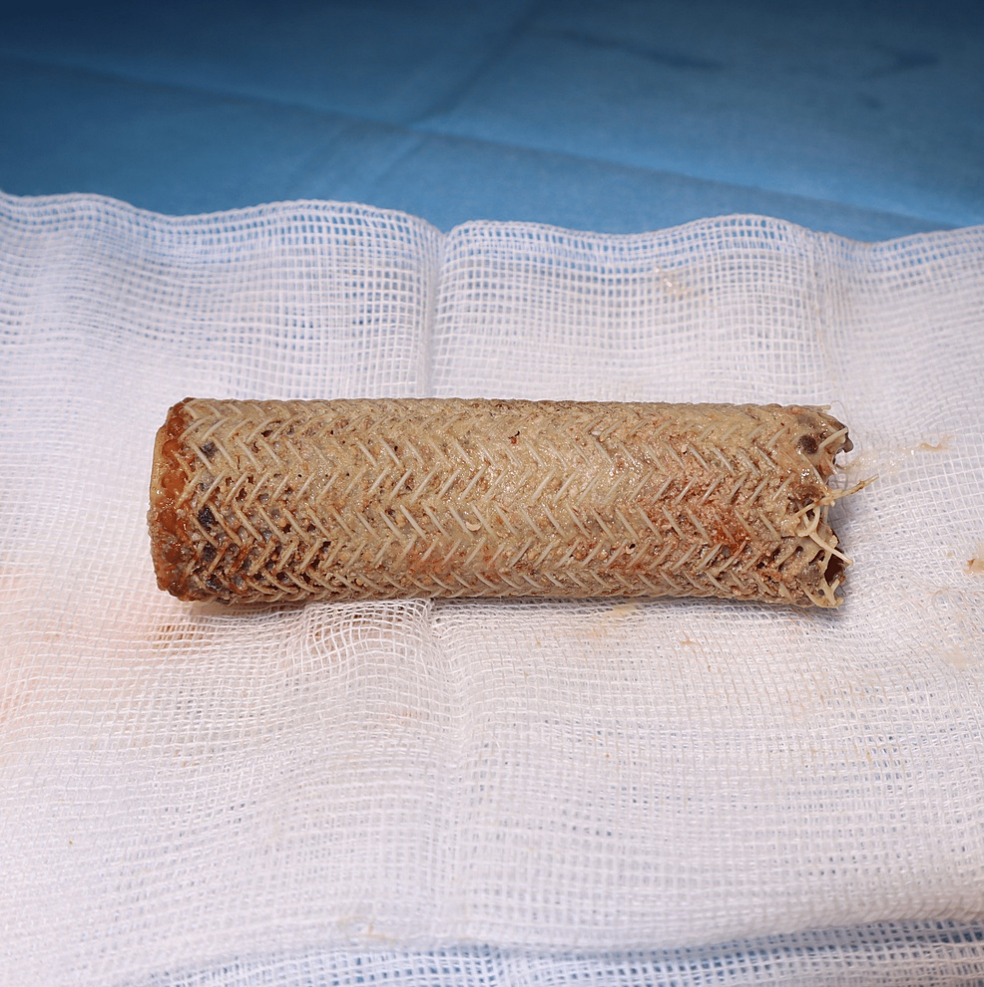
Appearance of the stent after endoscopic removal. It is completely intact except at the proximal end, which fragmented during the procedure.

The procedure was conducted under general anesthesia and curarization, with the assistance of an experienced anesthesiologist. After intubation with a Dumon rigid tracheoscope (outer diameter 13.20 mm, Efer-Dumon®, France), complete cleaning of the endobronchial secretions was performed. A progressive detachment of the stent from the tracheal wall was then carried out by means of a separation operated with the tip of the rigid tracheoscope. Subsequently, by grasping the stent with rigid forceps, an attempt was made to dislocate it proximally; however, the stent was very friable, detaching itself in fragments. The stent was then gently twisted using the same rigid forceps, reducing its proximal diameter and allowing its partial withdrawal inside the operating channel of the rigid tracheoscope. We then proceeded to the en-bloc removal of the tracheoscope and the partially recovered stent (Figure 3).

The patient was immediately reintubated with an 8.5mm endotracheal tube. No residual stent fragments were found within the airway. The procedure continued using flexible bronchoscopy, with the demolition by laser and diathermic loop of the granulomatous lesions localized at the level of the main bronchi, restoring their lumen. At the end of the procedure, 40mg of methylprednisolone was injected into the mucosa of the wall of the main bronchi. During the procedure, the decision was made not to place a new stent, as the lumen of the central airways was adequate and not excessively variable even during spontaneous ventilation evaluated at the end of residual curarization, and extubation. The patient underwent systemic corticosteroid and antibiotic therapy and was discharged five days after the procedure, scheduling an endoscopic check-up at 30 days, which confirmed the persistence of an optimal airway lumen.

## Discussion

Our case underlines the importance of timely and scrupulous follow-up of patients who undergo tracheal stent placement. The permanence of the stent in the case presented led to the formation of giant granulomas and the bacterial colonization of the stent itself, causing recurrent pulmonary infections, which led the patient to be hospitalized.

The patient presented here was lost to follow-up. We recommend that during each follow-up visit or after an endoscopic procedure, the outcome of the evaluation and the appointment for the next visit must be communicated to the patient and any caregiver. In adequately staffed centers, follow-up by telephone call, or employing telemedicine technologies may also be considered part of the care pathway. Furthermore, the knowledge of other medical professionals regarding the need to maintain scheduled check-ups on airway stents should not be taken for granted. The follow-up of cases similar to the one presented must be not only radiological but also bronchoscopic, as the inspection allows the early diagnosis of complications and their simultaneous management, regardless of the patient's symptomatic status [5].

We advise considering the structural alterations of the materials of a stent long held in place and anticipating the issues that may arise during the removal procedure, such as stent fragmentation and consequent injury to the central airways by their rupture, fissuring, or abrasion [6]. Rigid bronchoscopy allows complete control of the airway during the procedure and provides a large working channel. It is the gold standard for dealing with eventual massive bleeding and intraprocedural airway emergencies. In addition, it allows the use of large-sized endoscopic tools suitable for extracting and manipulating the stent [7]. A procedure such as the one presented in this document should not be performed under flexible bronchoscopy unless in conjunction with rigid bronchoscopy.

## Conclusions

Removal of stents left in place for a long time is a rare event; for this reason, a high level of experience of the operator and accurate preoperative planning ensure maximum patient safety. Six months after the procedure, the patient had no recurrence of lower respiratory tract infections and respiratory symptoms were adequately controlled.
